# *Ardisia crispa* roots inhibit cyclooxygenase and suppress angiogenesis

**DOI:** 10.1186/1472-6882-14-102

**Published:** 2014-03-19

**Authors:** Dayang Erna Zulaikha Awang Hamsin, Roslida Abdul Hamid, Latifah Saiful Yazan, Che Norma Mat Taib, Looi Ting Yeong

**Affiliations:** 1 Department of Biomedical Science, Faculty of Medicine and Health Sciences, Universiti Putra Malaysia, Serdang 43400, Selangor; 2 Department of Anatomy, Faculty of Medicine and Health Sciences, Universiti Putra Malaysia, Serdang 43400, Selangor; 3 Department of Paraclinical Science, Faculty of Medicine and Health Sciences, Universiti Malaysia Sarawak, Kuching 93150, Sarawak

**Keywords:** *Ardisia crispa*, COX inhibitor, LOX inhibitor, Soy lipoxygenase assay

## Abstract

**Background:**

In our previous studies conducted on *Ardisia crispa* roots, it was shown that *Ardisia crispa* root inhibited inflammation-induced angiogenesis *in vivo*. The present study was conducted to identify whether the anti-angiogenic properties of *Ardisia crispa* roots was partly due to either cyclooxygenase (COX) or/and lipoxygenase (LOX) activity inhibition in separate in vitro studies.

**Methods:**

Benzoquinonoid fraction (BQ) was isolated from hexane extract by column chromatography, and later analyzed by using gas chromatography–mass spectrometry (GC-MS). Anti-angiogenic effect was studied on mouse sponge implantation assay. *Ardisia crispa* ethanolic rich fraction (ACRH), quinone-rich fraction (QRF) and BQ were screened for COX assay to evaluate their selectivity towards two isoforms (COX-1 and COX-2), The experiment on soy lipoxygenase (LOX) inhibitory assay was also performed to determine the inhibitory effect of ACRH, QRF and BQ on soy LOX.

**Results:**

BQ was confirmed to consist of 2-methoxy-6-undecyl-1,4-benzoquinone, when compared with previous data. Antiangiogenesis study exhibited a reduction of mean vascular density (MVD) in both ACRH and QRF, compared to control. In vitro study showed that both ACRH and QRF inhibited both COX-1 and COX-2, despite COX-2 inhibition being slightly higher than COX-1 in BQ. On the other hand, both ACRH and QRF were shown to have poor LOX inhibitory activity, but not BQ.

**Conclusions:**

In conclusion, ACRH and QRF might possibly exhibit its anti-angiogenic effect by inhibiting cyclooxygenase. However, both of them were shown to possess poor LOX inhibitory activity. On the other hand, BQ displayed selectivity to COX-2 inhibitory property as well as LOX inhibitory effect.

## Background

Angiogenesis is a fundamental process of new capillary formation which is physiologically important in wound healing and reproduction [1]. Under normal physiologic circumstances, the body controls angiogenesis by producing a precise balance of pro-angiogenic and anti-angiogenic factors [2]. The imbalance of these factors will result in excessive or insufficient angiogenesis. Excessive angiogenesis for instance, contributes to initiation, progression, and prognosis of numerous diseases, such as cancer, arthritis, and cardiovascular diseases [3].

Cyclooxygenase (COX), also known as prostaglandin endoperoxide synthase, is a rate-limiting enzyme that catalyzes the transformation of arachidonic acid into prostaglandin H_2_ (PGH_2_), which eventually leads to the biosynthesis of other prostanoids (i.e. prostaglandins, prostacyclin and thromboxane) [4]. There are two isozymes identified in the COX family, which are COX-1 and COX-2. Whilst the COX-1 expression is constitutive in most tissue and exhibit physiological roles in the body, COX-2 expression is inducible upon a wide spectrum of stimuli, such as inflammatory responses [5].

Whilst COX-2 is important in catalyzing prostaglandin biosynthesis during inflammation, it also contributes to angiogenesis by upregulating VEGF level [6]. COX-2 is shown to be induced not only in the endothelial cells during inflammation, but also is overexpressed in tumour vasculatures [7]. Since COX-2 level is shown to be upregulated in preneoplastic condition as well as early cancer, it is suggested that COX-2 inhibition may prevent carcinogenic progression at several stages [8].

Meanwhile, lipoxygenase (LOX) converts arachidonic acid into various leukotrienes that play an important role in inflammation [9]. LOX involvement in angiogenesis is evident. For instance, 5-lipoxygenase (5-LOX) product, namely 5-HETE and LTA_4_, potently upregulate VEGF transcription in human malignant mesethelioma model [10]. Apart from that, since VEGF is a potent pro-angiogenic factor, 5-LOX was shown to promote *in vivo* tumour development by a direct proliferative stimulus on cancer cells and a potentiation of the pro-angiogenic response by the host stromal cells. 5-LOX products were also demonstrated to upregulate VEGF transcription in human umbilical vein endothelial cells [10]. Lipoxygenase metabolites were shown to enhance tumourigenesis, thus implying that the intervention through this pathway might be useful in arresting cancer progression [11].

*Ardisia crispa* has been reported in previous studies to exhibit anti- metastatic [12], anti-inflammatory, anti-hyperalgesic [13] and antiangiogenic [14] properties *in vivo*. Preliminary phytochemical studies conducted on *Ardisia crispa* reported the isolation of compounds such as triterpenoid saponins [15], and benzoquinones [12,16], which were shown to exert various properties including the aforementioned activities *in vivo*. This study aims to confirm the antiangiogenic activities exhibited by ACRH and QRF, and investigate whether the angiogenic activities shown are due to either cyclooxygenase or/and lipoxygenase activity inhibition.

## Methods

### Chemicals and drugs

Gelatine from cold fish, agarose, DMSO, phosphate-buffered saline (PBS) (Sigma Aldrich, USA), licofelone (Cayman, USA), gelatine sponge (Pharmacia and Upjohn, USA), histosec pastilles (Merck, Germany), Hematoxylin and Eosin (Merck, Germany), formalin (Merck, Germany), nylon suture 16 mm (Unik, USA), Burnolplus® (Reckitt Benckiser, USA), high profile microtome blade (Leica Microsystems, USA), coverslip (Hirschmann Laborgerate, Germany), DPX (BDH, USA), Colorimetric COX (ovine) Inhibitor Screening Assay Kit (No. 760111) (Cayman, USA), Soybean lipoxygenase type I-B, Linoleic acid, Sodium phosphate monobasic, Sodium phosphate dibasic (Sigma Aldrich, USA). All other reagents were of analytical grades and are therefore available commercially.

### Plant material

*Ardisia crispa* roots (ACR) was collected in Machang, Kelantam and identified by Dr. Roslida Abdul Hamid (Universiti Putra Malaysia). *Ardisia crispa* roots (ACR) with voucher specimen 20841 from Herbarium of Universiti Kebangsaan Malaysia was dried in an oven at 60°C for 5 days (Memmert, Germany) and grinded to form powder (Retsch, Germany). Soon after, ACR was extracted using 80% EtOH (v/v; 2000 mL × 3, 48 hr each) and fractionated using n-hexane (2000 mL × 3, 48 hr each), to yield *Ardisia crispa* roots ethanolic hexane fraction (ACRH), as proposed previously [17].

### Separation and isolation of benzoquinonoid fraction (BQ)

ACRH (6 g) was later separated by silica gel column chromatography using *n*-hexane and progressing to *n*-hexane/ethyl acetate (from 10:0 to 5:5, v/v) to give five fractions A-E. Fraction C and D (obtained from the mobile phase of *n*-hexane: EtOAc at 7:3 and 6:4) were pooled together, labelled as CD and was further rechromatographed with the same solvent system of *n*-hexane/EtOAc (from 9:1 to 5:5) to afford five subfractions CD1-CD5. Subfractions CD3 and CD4 were combined together, to yield a quinone rich fraction (QRF) (38.33%, w/w), which was compared with the reference, AC-2 [18] at the similar spots (R_f_: 0.76) using thin layer chromatography (TLC) technique, with chloroform as its developing system.

**Figure 1:**
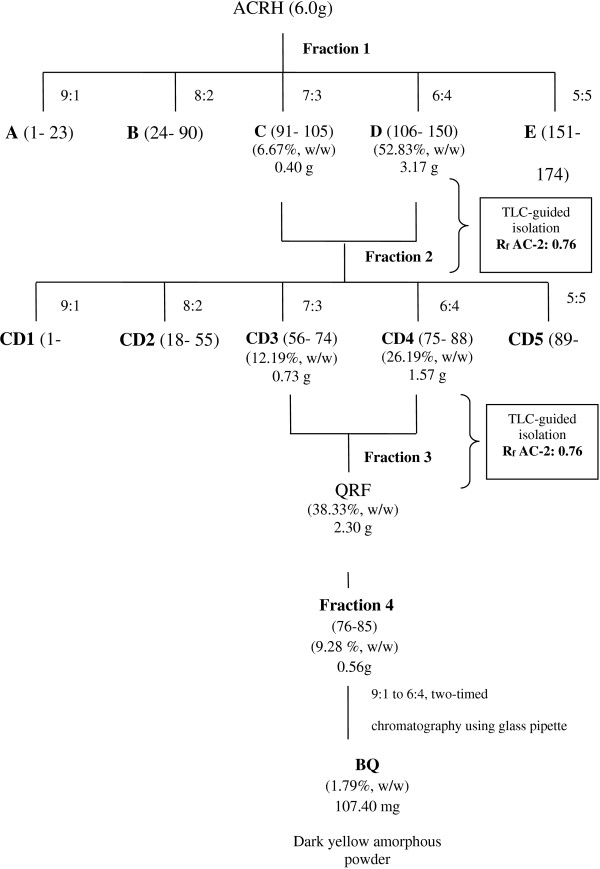
**TLC-guided chromatographic isolation of BQ from *Ardisia crispa *** **root.**

QRF was re-chromatographed using similar gradient of elution, hexane-EtOAc (10:0 to 6:4, 500 ml, v/v), affording four major sub-fractions. Among the four sub-fractions, vials 76–85 were shown to contain the most concentrated yellowish spot, which shared similar spot as the reference (R_f_: 0.76). Therefore, the fractions contained in vials 76 to 85 were pooled, and purified by washing the fraction with hexane to eliminate excess oil and debris. Re-chromatography using glass pipette was done two times using similar gradient of mobile phase (9:1 to 6:4; 10 ml, v/v) to yield approximately 107.4 mg of fraction labelled as benzoquinonoid fraction (BQ) (1.79%, w/w). BQ was characterized as a dark yellow amorphous powder. It was further resolved in GC-MS for the identification purpose. A flow chart portraying fractionation process and its yield was summarized in Figure 1.

### Experimental animals

Male ICR mice (25-30 g) at 6–8 weeks of age were used with each cage accommodated 6 mice in a standard laboratory condition (temperature 25 ± 2°C, and 12-hour light and dark cycle). The animals had access to water and food pellet *ad libitum*. Ethical clearance was obtained from the Animal Care and Use Committee (ACUC), Faculty of Medicine and Health Sciences, Universiti Putra Malaysia, Malaysia (Reference number: UPM/FPSK/PADS/UUH/F05).

### Mouse sponge implantation assay

This study was carried out according to previously proposed method [19]. Prior to experimentation, absorbable gelatin sponge was cut (5 mm × 8 mm) and hydrated in sterile PBS overnight at 4°C. Approximately 100 μl of 0.4% agarose was mixed with 50 μl of VEGF (2 ng/μl) and 50 μl of treatments (licofelone, ACRH or QRF) (2 ng/μl) or DMSO 1% (vehicle). The sponges were allowed to solidify at room temperature for one hour before commencement of the experiment. Mice were anesthetized with Avertin (10 mL/kg) prior to experimentation. Hardened gel piece was inserted into incised subcutaneous pocket created laterally. The animals were allowed to recuperate for 14 days. After that, the animals were euthanized and the gel piece was harvested and rinsed with normal saline.

### Histopathological evaluation

The sponge samples were fixed in 10% formalin (v/v) for 7 days and subjected to histopathological analysis. The number of vessels was counted in 15 consecutive fields using a 20*X* objective and the mean MVD was calculated [19].

### Cyclooxygenase inhibitory assay

Cyclooxygenase activity assay was performed according to Yang et al. [20], with slight modification. Inhibitory activity of ACRH, QRF and BQ towards COX-1 and COX-2 activity was determined by using colorimetric COX (ovine) inhibitor screening assay kit (Cayman, No. 760111). The assay was conducted by monitoring the appearance of oxidized N, N, N’, N’- tetramethyl-p-phenediamine (TMPD) at 590 mm. Aspirin served as positive control [20]. The test compounds were dissolved in 1% DMSO (v/v) at 5 different concentrations, which were 12.5, 25, 50, 100 and 200 μg/mL. The experiment was done in accordance to the one recommended by the supplier. The plate was shaken for a few seconds and incubated for 5 min at 25°C. 20 μl of the colorimetric substrate solution (TMPD) was added to all of the wells. 20 μl of arachidonic acid was added to all the wells. The plate was shaken for a few seconds and incubated for 5 min at 25°C. The absorbance was measured at 590 nm using a microplate reader. Average absorbance was calculated for all the samples (n = 3) in order to determine the percentage of inhibition. The calculation of inhibitory percentage is as portrayed below:

$$\begin{array}{cc} \%\mathrm{inhibition}= & \frac{\left(100\%\mathrm{initial}\;\mathrm{activity}‒\mathrm{inhibitor}\;\mathrm{wells}\right)}{100\%\mathrm{initial}\;\mathrm{activity}}\\ & \times 100 \end{array}$$

### Soy lipoxygenase inhibitory assay

Soy lipoxygenase inhibitory assay was performed according to the method proposed by Azhar-Ul-Haq et al. [21]. Rutin was used as positive control [22]. 160 μl of 100 mM sodium phosphate buffer (pH8.0), 10 μl of ACRH and QRF were dissolved in ethanol in different concentrations (200 μg/mL, 100 μg/mL, 50 μg/mL, 25 μg/mL, and 12.5 μg/mL) and 20 μl of lipoxygenase solution were mixed in the well and incubated for 10 mins at room temperature. The reaction was then initiated by adding 10 μl of linoleic solution and the absorbance was determined at 234 nm after 6 minutes. All concentrations were performed in triplicate. The IC_50_ value was then calculated.

### Statistical analysis

In mouse sponge implantation assay, statistical analysis was performed using SPSS version 16. One-way Analysis of Variance (ANOVA) with post-hoc test of Least Significant Difference (LSD) were utilised with P < 0.05 was considered to be statistically significant. The results were reported as mean ± SEM. For in vitro studies, average of the results obtained was determined in order to construct a graph, whereby IC_50_ of each groups were identified, respectively. IC_50_ was calculated using GraphPad Prism 5 software. Data were reported as mean ± SEM.

## Results and discussions

### Extraction and separation of quinone rich fraction (QRF) and benzoquinonoid fraction (BQ)

Approximately 500 g of *Ardisia crispa* root plant material extraction yielded 21.01 g (25.87%, w/w) of *Ardisia crispa* root hexane fraction (ACRH), characterized as a brown-black oily mass. 6 g of ACRH was eluted through a column to yield 2.3 g of QRF (38.38%, w/w), using solvent hexane: ethyl acetate with ratio of 7:3 and 6:4 (500 mL; each mobile phase ratio). By using AC-2 (reference compound) as guidance in the TLC-guided fractionation, refractionation of QRF was carried out to pool benzoquinonoid fraction, yielding 107.4 mg of benzoquinonoid compound (1.79%, w/w) with similar chemical characterization as 2-methoxy-6-undecyl-1.4-benzoquinone [16], labeled as BQ. Isolating benzoquinone has been the basis of the isolation protocol of this study, primarily as unusual series of benzoquinones isolated from various species of *Ardisia crispa*, such as *Ardisia sieboldii*[23] and *Ardisia japonica*[18] were shown to possess anti-inflammatory properties, and anti-inflammation is shown to be strongly correlated to anti-angiogenesis [24].

### Chromatographic analysis of BQ

In the present study, GC-MS method was employed to confirm the nature of the AC-2 (reference) as a 1, 4-benzoquinone derivative, specifically 2-methoxy-6-undecyl-1, 4-benzoquinone, which was first isolated by Roslida [16]. The gas chromatograms of BQ were depicted in Additional file 1. One major peak (Peak 2), assumed to be 2-methoxy-6-undecyl-1,4-benzoquinone was shown at 39.54 min. Whilst, minor peaks at 35.51 (Peak 1), 41.79 (Peak 3) and 49.92 min were also displayed in the spectrum. The major peak at 39.537 min was further resolved for mass spectrometry, and compared with the reference [16] (Additional file 2: a & b)

The molecular ion peak of chromatogram present at min 39.537 for BQ was 292.1 [M+], with mass break down also present at m/z 193.1, 179.1, 154, 124, and 69 (Additional file 2: a). Its molecular ion peak of the chromatogram present was consistent with that of previously obtained in previous report (292.0 [M+]) (Additional file 2: b), thus suggesting the compound identified as 2-methoxy-6-undecyl-1,4-benzoquinone.

### Mouse sponge implantation method

Rat air pouch [25] and Miles assay [26] were previously used to quantify angiogenesis and evaluate anti-angiogenic agents of ACRH and QRF *in vivo*[14]. The objective of using subcutaneous implant model was to trap angiogenic factor into a suitable carrier such as sponge-like structure, which will cause the recruitment of new blood vessels into implant. The increase of number of blood vessels present in the sponge, which originally contained none, will represent neovascularisation [27]. VEGF was used as the angiogenic factor as it is a potent vasculogenic agent [28]. This assay offered some distinct advantages over conventional angiogenic assays, such as biocompatibility and the feasibility of studies conducted in a long term. Additionally, grafts can be implanted in immunologically normal rodents [19].

The anti-angiogenic effect of ACRH and QRF on mean vascular density (MVD) was determined in the present study. There was no significant difference in MVD of VEGF control (VEGF alone) and vehicle control (VEGF and DMSO), which also indicated DMSO as a vehicle did not possess any significant blood vessel- reducing activity in this study. However, treatment with licofelone (100 ng), ACRH (100 ng), and QRF (100 ng) resulted in a significant reduction in mean vascular density (MVD), with the MVD value being 3.67 ± 0.843 (P < 0.01), 5.00 ± 0.894 (P < 0.05), 2.83 ± 0.833 (P < 0.01), respectively, compared to VEGF control (7.76 ± 2.34). In addition, there was no significant difference observed in ACRH and QRF treatment group, compared to licofelone. The MVD of the treatment of ACRH and QRF at similar dose (100 ng), was also observed not to significantly differ as well. Interestingly, QRF is shown to be more potent than licofelone (Table 1).

**Table 1:** Quantification of mean vascular density (MVD) following various treatments in mouse sponge implantation assay

**Treatment**	**Mean vascular density (MVD) ± SEM**	**% inhibition**
VEGF	11.17 ± 2.442	-
VEGF + 1% DMSO (vehicle)	7.76 ± 2.348	-
VEGF + licofelone	3.67 ± 0.843^b^	67.14
VEGF + ACRH	5.00 ± 0.894^a^	55.23
VEGF + QRF	2.83 ± 0.833^b,A^	74.66

Data were presented as Mean ± SEM. Values with different superscript letters within the same column are statistically different ^a^P < 0.05; ^b^P < 0.01 compared to control group (VEGF), while ^A^P < 0.05 compared to control group (VEGF + 1% DMSO), determined by one-way ANOVA and LSD post-hoc test.

**Figure 2:**
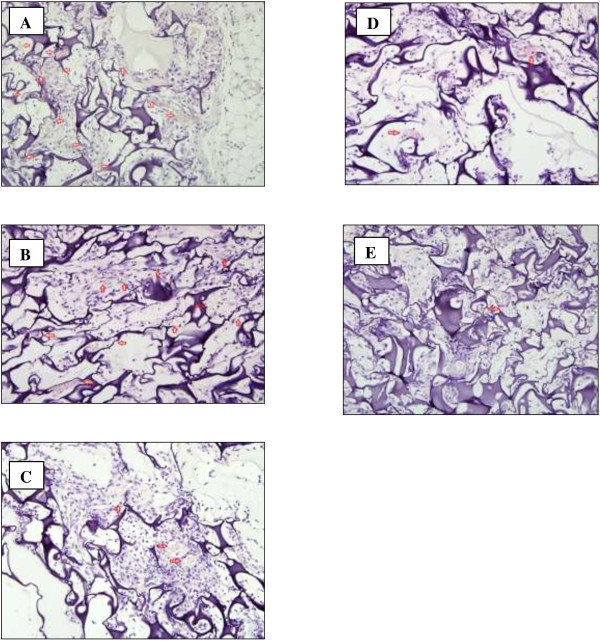
**Microphotograph of sections showing blood vessels around gels impregnated with (A) VEGF; (B) VEGF and DMSO; (C) VEGF and licofelone; (D) VEGF and ACRH; (E) VEGF and QRF. **Red arrows indicated the blood vessels (Magnification 200×).

High MVD would depict a dense blood vessels impregnated in the gel, while low MVD would show sparse blood vessels embedded in the gel. Histopathological slides of the gel sections for different treatments showing formation of blood vessels were shown in Figure 2A-E. It was noted that at 200× magnification, a dense blood vessels, which was also reflected in the MVD count, was found in the VEGF-impregnated gel and in VEGF-impregnated gel with DMSO (Figure 2A, B). Meanwhile, in the group of VEGF-embedded gel treated with licofelone, ACRH and QRF, scanted blood vessels were observed in the histological slide (Figure 2C-E).

### Cyclooxygenase inhibitory assay

Arachidonic acid is metabolized by cyclooxygenase and lipoxygenase pathways to form mediators such as prostaglandins and leukotrienes with significant roles in inflammatory response [29]. COX-2 and COX-2- derived prostaglandins have a significant role in angiogenesis in such a way that COX-2 antagonists have the ability to block neovascularisation [30]. In the present study, the inhibitory effects of ACRH, QRF and BQ to both COX isoforms (COX-1 and COX-2) were evaluated in vitro. Aspirin, a non-selective cyclooxygenase inhibitor was shown to inhibit both COX-1 and COX-2 competitively, which was consistent a previous study [20]. Among all NSAIDs, aspirin is a unique non-selective irreversible COX inhibitor due to its ability to acetylate the Ser530 hydroxyl group in the primary active site of COX-1 and COX-2 [31]. Celecoxib, a known selective COX-2 inhibitor, was shown have a good selectivity towards inhibition of COX-2 instead of COX-1. Celecoxib was introduced as a highly selective COX-2 inhibitor and is known as a remarkable anti-inflammatory drug with lower gastrointestinal complication [32]. In contrast, ACRH and QRF were shown to inhibit both COX-1 and COX-2 competitively; though COX-2 inhibition was slightly prominent. Interestingly, BQ was shown to possess selectivity towards COX-2 inhibition, and the action was comparable to celecoxib. The anti-angiogenic effect of ACRH and QRF could be partially explained by the potent inhibitory effect of compounds consisted in both ACRH and QRF on arachidonic metabolism through COX-2 pathway; though it is shown that both ACRH and QRF non-selectively inhibit COX-1 as well. It is also possible that BQ, which is a major chemical constituent of ACRH and QRF [14,16], is responsible in exerting the anti-angiogenic effect, mainly via COX-2 inhibition, based on the present data obtained.

ACRH and QRF were assessed in vitro to investigate their possible effects in COX activity inhibition. Percentage of inhibition of both isoforms of COX (COX-1 and COX-2) was found to be dose-dependent. Aspirin was shown to be the most effective COX-2 inhibitor with the lowest IC_50_ (26.69 ± 2.245 μg/mL), followed by celecoxib (29.10 ± 1.878 μg/mL), ACRH (37.79 ± 1.843 μg/mL) and QRF (35.24 ± 1.958) respectively. Aspirin displayed non-selectivity towards COX by inhibiting both isoforms competitively.

Meanwhile, celecoxib displayed more selectivity towards COX-2 than COX-1 with approximately 2-fold percentage of inhibition increase, notably in the concentration of 25 μg/mL and above. ACRH and QRF showed approximate result, as both ACRH and QRF did not display specific selectivity towards COX-2 inhibition. Both COX-1 and COX-2 were inhibited competitively, though COX-2 inhibition was more prominent, especially in high concentration (100 μg/mL and above). The pattern of COX-1 and COX-2 inhibition in ACRH and QRF was comparable to aspirin. However, BQ showed preference to COX-2 inhibition, with a 2.1-fold inhibition of COX-2 compared to inhibition of COX-1. The result was comparable to that of celecoxib. IC_50_ of both COX-1 and COX-2 following treatments were shown in Table 2. The percentage of inhibitions of COX isoforms following various treatments were also tabulated in Table 3.

**Table 2:** **IC**_**50 **_**of different treatments in cyclooxygenase inhibitory assay**

**Treatment**	**IC**_**50 **_**(μg/ml)**	
	**COX-1**	**COX-2**
Aspirin	43.93 ± 1.471	26.69 ± 2.245
Celecoxib	44.21 ± 2.757	29.10 ± 1.878
ACRH	29.57 ± 2.188	37.79 ± 1.843
QRF	21.44 ± 7.533	35.24 ± 1.958
fAC2 (Rich AC-2)	51.48 ± 2.599	33.00 ± 2.010

IC_50_ was reported as IC_50_ ± SEM with COX inhibitory effects determined in the concentration of 12.5, 25, 50, 100, and 200 μg/ml of each treatments priorly.

**Table 3:** Percentage of inhibitions of COX-1 and COX-2 following treatments

**Concentration (μg/ml)**	**Percentage of COX inhibition (%)**									
	**COX-1**					**COX-2**				
	**12.5**	**25**	**50**	**100**	**200**	**12.5**	**25**	**50**	**100**	**200**
Aspirin	27.778 ± 0.505	36.027 ± 1.347	37.879 ± 0.842	40.067 ± 1.347	46.465 ± 0.505	33.896 ± 1.074	51.380 ± 0.154	54.294 ± 0.307	59.049 ± 0.153	62.423 ± 0.154
Celecoxib	21.212 ± 1.010	23.569 ± 1.347	27.441 ± 1.852	31.818 ± 2.189	32.492 ± 1.179	25.920 ± 0.460	48.160 ± 0.614	52.454 ± 0.614	55.675 ± 1.074	63.650 ± 0.767
ACRH	26.094 ± 0.505	32.492 ± 0.505	34.512 ± 0.169	37.037 ± 0.000	38.215 ± 0.505	25.767 ± 1.534	38.037 ± 0.920	45.399 ± 0.614	49.693 ± 1.227	56.595 ± 0.153
QRF	25.589 ± 3.367	34.848 ± 0.506	35.690 ± 0.674	36.869 ± 2.189	37.710 ± 0.674	29.908 ± 0.460	41.258 ± 0.460	49.693 ± 0.000	51.687 ± 0.460	58.129 ± 0.154
BQ	20.707 ± 0.505	21.380 ± 0.169	25.253 ± 0.337	27.273 ± 0.337	29.125 ± 0.169	27.301 ± 0.000	43.558 ± 1.688	49.233 ± 0.154	57.209 ± 0.921	61.350 ± 1.277

Each concentration in its respective treatment groups were tested for its cyclooxygenase inhibitory activity (COX-1 and COX-2) in triplicate (n = 3).

### Soy lipoxygenase inhibitory assay

The use of soybean lipoxygenase in the in vitro inhibition study served as an appropriate model for the screening of plants with anti-inflammatory properties [31], as lipoxygenase found in plant was considered equivalent of the angiogenic cascades in animals [22]. ACRH and QRF did not display significant LOX inhibitory activity. The finding was in contrast with previous literature, as ACRH and QRF were shown to have anti-oxidant activities (unpublished data), and anti-oxidants were known to inhibit plant lipoxygenases [32]. Meanwhile, BQ displayed LOX inhibitory activity, which was also shown to be dose-dependent. Other than 1, 4-benzoquinone compound of *Ardisia crispa* roots shown in the present study, there are also several other 1, 4- benzoquinones isolated from other *Ardisia* species possessing potent 5-lipoxygenase inhibitory activity which were reported in previous studies, such as Ardisiaquinone A, isolated from *Ardisia sieboldii*[23], and Ardisianones A and B, isolated from *Ardisia japonica*[33]. Ardisiaquinone A for instance, was shown to decrease allergen-induced vascular permeability significantly in guinea pigs [34].

LOX-suppressive effect of ACRH, rich fraction and BQ were assessed in vitro. The inhibitory effect of soy lipoxygenase was found to be prominent in rutin (IC_50_: 44.37 ± 2.65 μg/mL). ACRH showed minimum LOX inhibition, with IC_50_ of 304.5 ± 0.32 μg/mL. Apart from that, QRF was also shown to be a weak inhibitor of LOX, with IC_50_ being 95.86 ± 2.17 μg/mL. BQ was found to be a moderate LOX inhibitor, with IC_50_ was found to be 66.65 ± 2.71 μg/mL. The IC_50_ of each treatment was depicted in Table 4. Meanwhile, the percentage of inhibition of soy lipoxygenase following different treatments was displayed in Figure 2.

**Table 4:** **IC**_**50 **_**of different treatments in soy LOX inhibitory assay**

**Treatment**	**Lipoxygenase assay (IC**_**50**_**)**
	**(μg/ml)**
Rutin	44.37 ± 2.65
Aspirin	137.4 ± 52.30
ACRH	304.5 ± 0.32
QRF	95.86 ± 2.17
BQ	66.65 ± 2.71

IC_50_ was reported as IC_50_ ± SEM with LOX inhibitory effects determined in the concentration of 12.5, 25, 50, 100, and 200 μg/ml of each treatments priorly.

Nonetheless, since angiogenesis is a multi-cellular event, it is important to further confirm anti-angiogenic activities of ACRH and QRF utilising various in vitro and *in vivo* models that include other prominent features of angiogenesis such as the endothelial proliferation, migration and tube formation [35]. Apart from that, the role of BQ specifically in eliciting COX-2 and LOX inhibitory responses in both in vitro and *in vivo* angiogenic models can be further elucidated, as it might become useful in the design of new drug which targets converging pathway of COX-2 and LOX.

## Conclusions

Taking the results together, it is confirmed that ACRH and QRF exhibited antiangiogenic properties by significantly reduce blood vessel formation, and it is possible that the mechanism of blood vessel reduction is partly mediated by non-selective inhibition of cyclooxygenase pathway. Through the COX and LOX inhibitory assays conducted, it is demonstrated that BQ exhibited promising inhibitory activities of selective COX-2 and LOX.

## Competing interests

The authors declare that they have no competing interest.

## Authors’ contributions

DEZAH performed the research and wrote the manuscript. RAH contributed to the experimental design, data interpretation, editing and submission of the manuscript. LSY contributed to the experimental design and data interpretation. CNMT contributed to the experimental design. YLT performed part of the research and its data interpretation. All authors read and approved the final manuscript.

## Pre-publication history

The pre-publication history for this paper can be accessed here:

http://www.biomedcentral.com/1472-6882/14/102/prepub

## Supplementary Material

Additional file 1 Gas chromatogram of BQ separated using gas chromatography technique. Click here for file

Additional file 2 **Mass-spectrum of (a) major compound in BQ at R**_**t**_**=39.537, and (b) the reference, 2-methoxy-6-undecyl-1,4-benzoqunone [**[23]**].** Click here for file
