# Supplementary material for: Ardisia crispa roots inhibit cyclooxygenase and suppress angiogenesis
Source: BMC Complement Altern Med. 2014 Mar 19;14:102. doi: 10.1186/1472-6882-14-102 (PMC4000009; doi:10.1186/1472-6882-14-102)
Supplement: Additional file 1 — Gas chromatogram of BQ separated using gas chromatography technique. [file 1472-6882-14-102-S1.doc]

**Additional file 1 – Gas chromatogram of BQ separated using gas chromatography technique.**

Peak 2 at R_t_=39.537 was found to be compatible with 2-methoxy-6-undecyl-1, 4-benzoquinone based on its similar molecular ion peak and consistent mass fragmentation


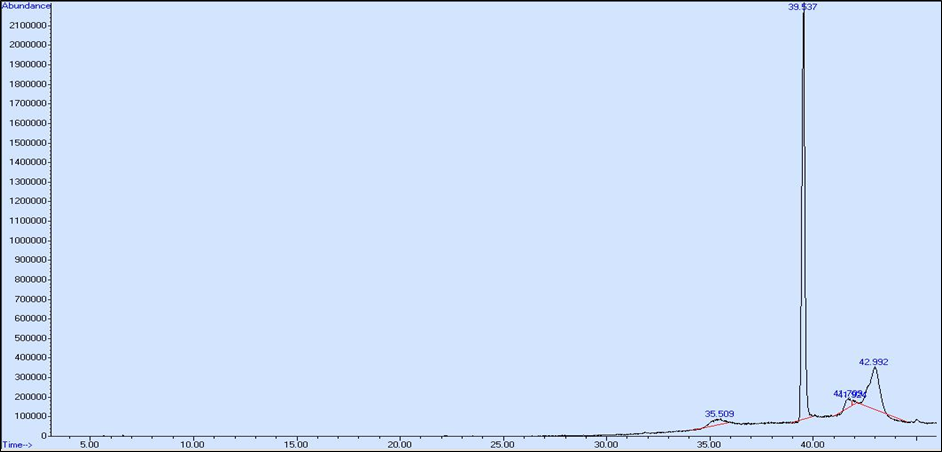


1

2

3

4

Peak 2 at Rt=39.537 was found to be compatible with 2-methoxy-6-undecyl-1, 4-benzoquinone based on its similar molecular ion peak and consistent mass fragmentation
