# Supplementary material for: Ardisia crispa roots inhibit cyclooxygenase and suppress angiogenesis
Source: BMC Complement Altern Med. 2014 Mar 19;14:102. doi: 10.1186/1472-6882-14-102 (PMC4000009; doi:10.1186/1472-6882-14-102)
Supplement: Additional file 2 — Mass-spectrum of (a) major compound in BQ at R t =39.537, and (b) the reference, 2-methoxy-6-undecyl-1,4-benzoqunone [[23]]. [file 1472-6882-14-102-S2.doc]

## Additional file 2 – Mass-spectrum of (a) major compound in BQ at R_t_=39.537, and (b) the reference, 2-methoxy-6-undecyl-1,4-benzoqunone [23]

(a)


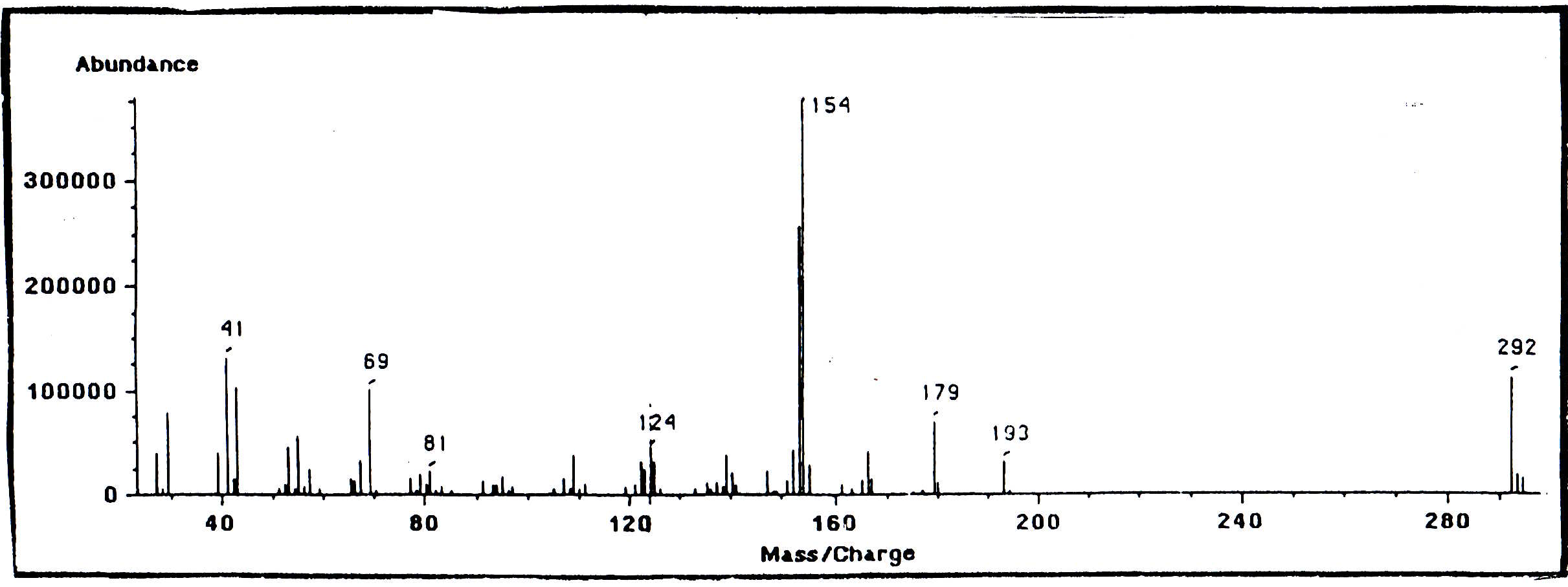

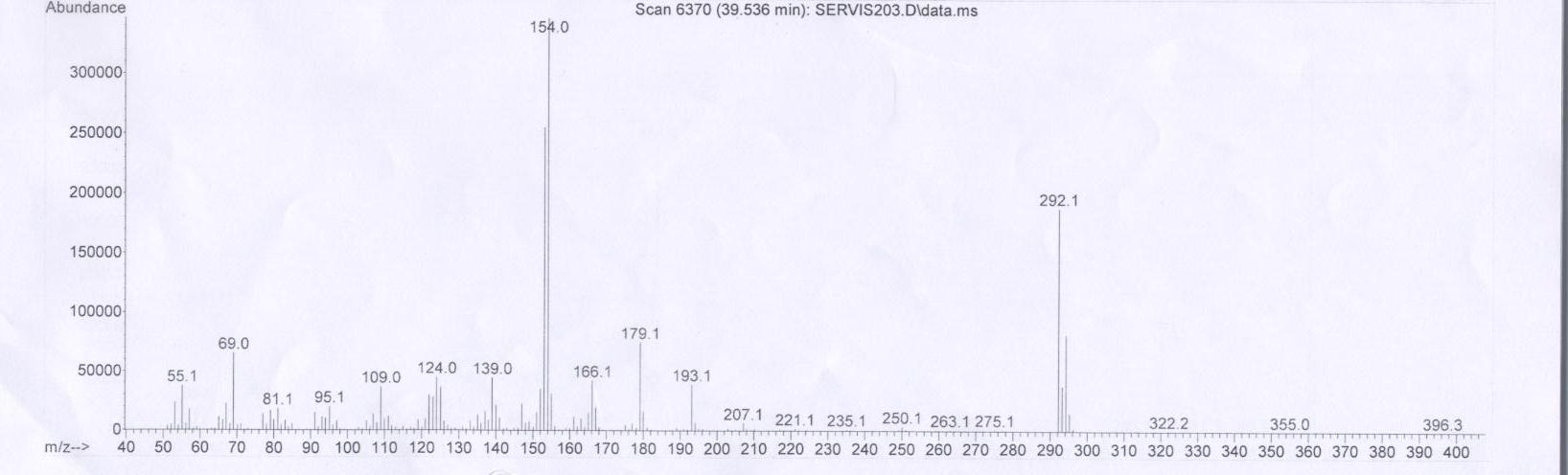


(b)
